# Adenosine, bridging chronic inflammation and tumor growth

**DOI:** 10.3389/fimmu.2023.1258637

**Published:** 2023-10-31

**Authors:** Luxia Chen, Mohamad Alabdullah, Karsten Mahnke

**Affiliations:** Department of Dermatology, University Hospital Heidelberg, Im Neuenheimer Feld, Heidelberg, Germany

**Keywords:** adenosine, tumor, chronic inflammation, immunosuppression, hypoxia

## Abstract

Adenosine (Ado) is a well-known immunosuppressive agent that may be released or generated extracellularly by cells, via degrading ATP by the sequential actions of the ectonucleotides CD39 and CD73. During inflammation Ado is produced by leukocytes and tissue cells by different means to initiate the healing phase. Ado downregulates the activation and the effector functions of different leukocyte (sub-) populations and stimulates proliferation of fibroblasts for re-establishment of intact tissues. Therefore, the anti-inflammatory actions of Ado are already intrinsically triggered during each episode of inflammation. These tissue-regenerating and inflammation-tempering purposes of Ado can become counterproductive. In chronic inflammation, it is possible that Ado-driven anti-inflammatory actions sustain the inflammation and prevent the final clearance of the tissues from possible pathogens. These chronic infections are characterized by increased tissue damage, remodeling and accumulating DNA damage, and are thus prone for tumor formation. Developing tumors may further enhance immunosuppressive actions by producing Ado by themselves, or by “hijacking” CD39^+^/CD73^+^ cells that had already developed during chronic inflammation. This review describes different and mostly convergent mechanisms of how Ado-induced immune suppression, initially induced in inflammation, can lead to tumor formation and outgrowth.

## 1 Introduction

A connection between inflammation and cancer was already reported in 1863 by Rudolf Virchow (1). Recent epidemiological studies have highlighted the interplay between cancer and inflammation, whether it is triggered by infection or not. Two major hypotheses have been proposed to explain the potential association between inflammation and cancer. One hypothesis implicates that sustained and pathogenic inflammation intrinsically promotes genetic instability during cancer pathogenesis. In another hypothesis, a defective host immunity, which is unable to clear pathogens, leads to chronic inflammation that finally facilitates cancer development (2, 3). These two interconnected pathways result in immunosuppression, thereby providing a favorable tissue environment for tumor development.

Immunosuppression is ubiquitously present in healthy and diseased individuals. It is a critical mechanism for maintaining self-tolerance and for the resolution of acute inflammation. At later stages of a ceasing inflammation, immunosuppression facilitates tissue remodeling and repair. On the contrary, immunosuppression is also a mechanism by which pathogens and tumor cells escape immune surveillance to survive and to proliferate.

Adenosine (Ado), besides being a neurotransmitter, has been thoroughly investigated for its immunosuppressive functions. Ado is generated from the sequential hydrolysis of adenosine triphosphate (ATP) by the ectonucleotidases CD39 and CD73, or by release through pores. Cells which express CD39 and CD73 exert suppressive function through the production of Ado. For instance, regulatory T cells (Tregs) constitutively express CD73, and their suppressive capacity in several inflammatory models depends on the production of Ado. In the immune system Ado is capable of suppressing dendritic cells, T cells, B cells and monocytes in a way that these cells are impeded in different immune stimulatory functions. This immunosuppressive activity of Ado is mainly mediated by A_2_A and A_2_B Ado receptors, however, A1 and A3 receptors for Ado are also defined but their cellular signaling and contribution to immune suppression is less clear.

Ado may have implications for the development of tumors from chronic inflammations. Due to its regulatory functions during inflammation, Ado may at first maintain an ongoing immune reaction by preventing the immune system from finally clearing pathogens or harmful agents from the body, thus helping to turn an acute into a chronic inflammation. Such a lingering inflammation provides a tissue environment that fosters DNA damage and neoplasia, eventually leading to tumor formation. The developing tumors start growing, and an already adenosine-harboring and thus immune suppressed tissue, is less capable of preventing the outgrowth of tumors. Moreover, some tumors even express the Ado producing enzymes CD39 and CD73 themselves, or are able to recruit further Ado-producing cells to create a tumor permissive environment. Although not many data on the detailed mechanisms are available yet, a role for Ado produced in inflamed tissues for later tumor development is conceivable, given its strong immune suppressive properties.

## 2 How is tumor growth, chronic inflammation and Ado connected at all?

### 2.1 Molecular mechanisms

Many factors and molecular means are involved in cancer initiation, among them are inflammation and infection. Between 15% and 20% of all neoplasms are thought to be initiated by infections, chronic inflammation or autoimmune inflammatory disease (4, 5). Among them are colorectal carcinoma, occurring with high prevalence in persons suffering from Inflammatory bowel diseases, such as Crohn’s disease and chronic ulcerative colitis, (6, 7), gastric cancer that is induced by Helicobacter pylori-infections (8), and human papillomavirus-related cervical cancer (9). Moreover, patients have an increased risk of pancreatic cancer when suffering from chronic pancreatitis (10), and lung cancer is enhanced by chronic lung infections, such as tuberculosis (11).

**Figure 1:**
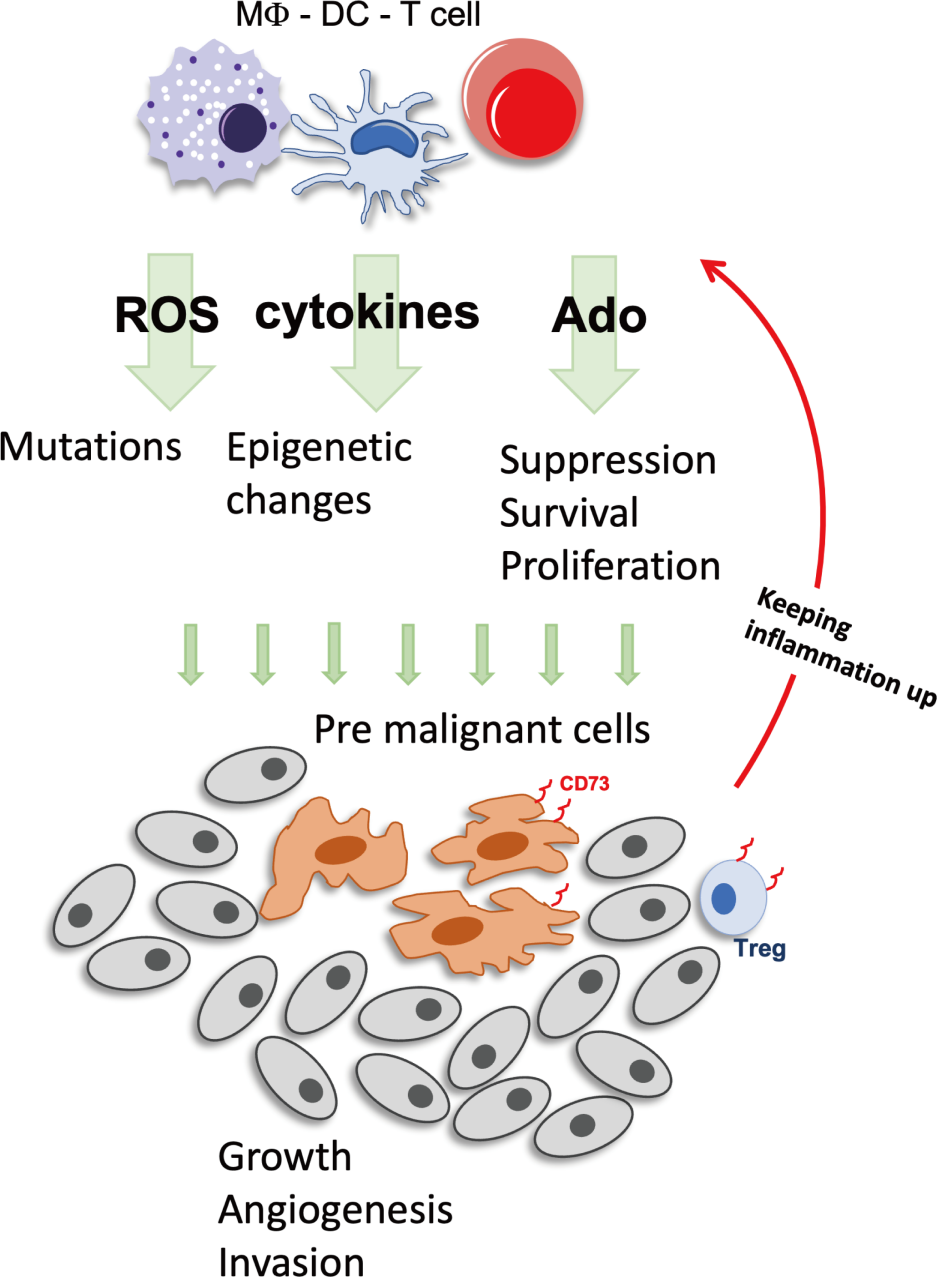
Inflammation can foster tumor development. During inflammation Immune cells produce factors such as ROS, cytokines and Ado that stimulate cell growth and battle pathogens. But these factors have also mutagenic potential and once premalignant cells have developed, the immunosuppressive actions will be augmented by either direct production of Ado, expression of CD73^+^, or by recruitment of CD73^+^ cells, enabling the tumor to create a favorable growth environment. Ado, adenosine; DC, Dendritic cell; MΦ, macrophage; ROS, reactive oxygen species; Treg, regulatory T cells.

An inflammatory microenvironment is believed to raise mutation rates and to promote the proliferation of mutated cells. Inflammatory cells generate reactive oxygen species (ROS) and reactive nitrogen intermediates, causing DNA damage and genomic instability (**Figure 1**). For example, ROS has been shown to directly deactivate mismatch repair enzymes (3, 12). And once the mismatch repair system is compromised, inflammation-driven mutagenesis intensifies, leading to the inactivation of crucial tumor suppressors like transforming growth factor β (TGFβ) receptor type 2 (Tgfbr2) and Bcl-2 Associated X protein (Bax) (3). p53 mutations that are also likely to result from oxidative damage during inflammation, have been detected in both, cancer cells and non-dysplastic inflamed epithelium, in colitis associated cancer, further substantiating the notion that chronic inflammation induces genomic changes (13).

In more general terms chronic inflammation may act as potent co-factor for tumor development, as the colonic irritant dextran sodium sulfate (DSS) may lead to DNA damage and the development of colonic adenomas when given during chronic inflammation (14). In contrast, DSS alone is only a weak carcinogen and is not able to induce tumors in “healthy” subjects by itself (15).

Another link between inflammation and oncogenic mutations involves the upregulation of AID (activation-induced cytidine deaminase), an enzyme that induces cytosine deamination in DNA during immunoglobulin gene class switching (16). AID is overexpressed in various cancers, and it is induced by inflammatory cytokines through NF-κB-dependent mechanisms or TGFβ (16). AID promotes genomic instability and increases mutation occurrence during the error-prone joining of DNA breaks, impacting critical cancer genes like Tp53, c-Myc, and Bcl-6 (3). AID contributes to the development of lymphomas, gastric cancers, and liver cancers (16, 17). Other suggested mechanisms of inflammation-induced mutagenesis involve effects on non-homologous recombination and NF-κB-mediated inactivation of p53-dependent genome surveillance (3). Additionally, inflammation has been connected to epigenetic reprogramming through Jmjd3 (Jumonji domain-containing protein D3), an NF-κB target gene (18).

### 2.2 The role of leukocytes and adenosine

Although all of these aforementioned tumors originate from different tissues, and are associated with different types of infection, and may employ different molecular pathways for tumor development, a common denominator may be the recruitment of immune cells during the onset of the Inflammation and/or the following tumor growth.

The consecutive infiltration of the tissues by immune cells is initially designed to battle bacteria, viruses or other harmful agents. To this end, the onset of an inflammatory episode helps to clear the body from the infection, and later, immune cells help to downregulate inflammation and to promote healing and the re-establishment of intact tissues. For these later tasks, immune cells are capable of producing immunosuppressive mediators and growth factors, which are meant to repair tissue damage and to stimulate proliferation of otherwise quiescent cells that are adjacent to the site of infection.

One of these factors is the broadly expressed suppressive mediator Ado. It can be released by cells, or it is extracellularly produced by actions of the two ectonucleotidases CD39 and CD73. These enzymes are expressed on various types of immune cells, e.g. T cells, dendritic cells, B cells and neutrophils, with a preference for immune suppressive cells, such as regulatory T cells, immature dendritic cells and suppressive B cells (19). As for their function, CD39 dephosphorylates proinflammatory extracellular ATP that is released by dying, injured or alarmed cells, a situation that occurs during inflammation, to Ado diphosphate (ADP) and Ado monophosphate (AMP). In a second step, AMP can be degraded to Ado, which has, as opposed to ATP, potent anti-inflammatory potential. Ado engages four G protein-coupled adenosine receptors (ARs), e.g. A_1_, A_2_A, A_2_B and A_3_, and activates downstream signaling pathways, modulating various cellular functions according to different cell types and receptor expression patterns (20). A_2_A and A_2_B are predominantly involved in the immunosuppressive function of Ado (21), and became the focus of many studies.

**Figure 2:**
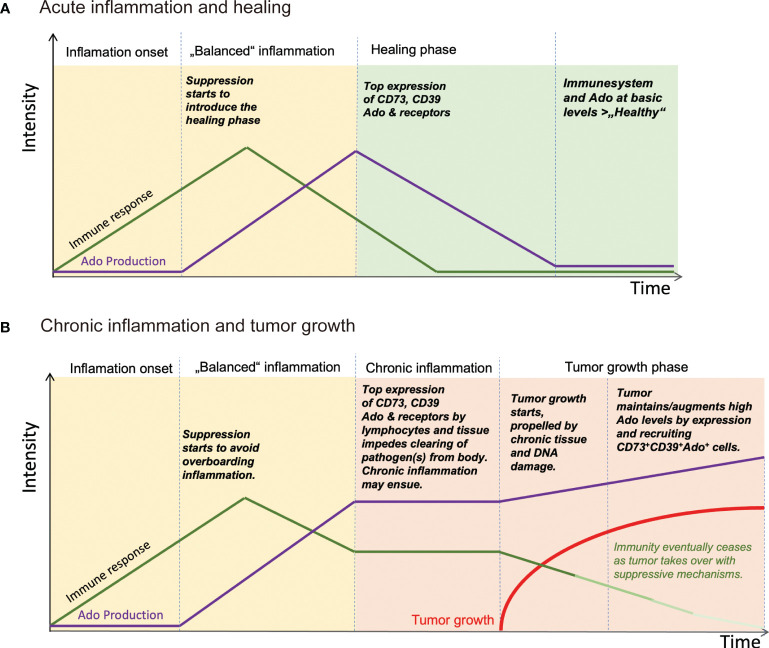
Schematic view of how Ado levels are involved in regulating inflammation and stimulating tumor growth. **(A)** After insult and pathogen invasion, immune response is started. Soon thereafter Ado is produced by leukocytes, for example regulatory T cells and tissue cells, to dampen the immune reaction and to start the healing phase of an infection. The infection ceases and the tissue is regenerated with help of Ado and other immune suppressive mediators. **(B)** In the course of an infection, the immune suppressive effects of Ado together with an ongoing immune response may be too strong to be cleared at an instant. A chronic inflammation may ensue with high levels of Ado. Ado is creating an immunosuppressive tissue environment and at the same time inflammation induces mutagenesis, eventually leading to development of tumors. Once established, tumors may recruit Ado producing cells or generate Ado by themselves, maintaining an immunosuppressive environment, to escape immune surveillance. Ado: adenosine.

Thus, an inherent immunosuppressive and even pro-proliferative function of immune cells, owed to their capability to produce Ado, or to react to it, is already present during inflammatory episodes, and tumors, early on during development of cancer, may take advantage of this to escape immunologic control. In a broader sense, actions of Ado during chronic inflammation is tumorigenic whilst anti-inflammatory Ado can maintain neoplasms and growth (**Figure 2**). The specific time sequence of Ado-related signaling can confer differential effects on tumorigenesis or cancer progression via several mechanisms on distinct cell types as we will further discuss.

## 3 Functions of Ado during inflammation and tumor development

### 3.1 Direct pro-inflammatory effects of Ado

It has been delineated in many publications that during infection the incoming innate immune cells, and later, cells from the adaptive immune system, may have a great impact for “preparing the soil for tumor growth”. That is, continuous inflammation with cell death, tissue destruction and enhanced cell proliferation may foster an environment in which gene editing, modification of DNA and proliferative pathways (e.g. NF-κB, and Wnt signaling), common to inflammation and cancerous cells, may lead to tumor development (22–24). In this regard some reports show direct proinflammatory actions of Ado.

In a model of DSS-induced colitis blockade of A_2_B Ado receptors by the antagonist ATL-801 reduced the severity of colitis, along with lower levels of IL6 (25). Similarly, PSB1115, an antagonist for A_2_B Ado receptors, suppressed the inflammation of the intestine in a neonatal rat model of enterocolitis (26). These studies were further supported by the observation that genetic deletion of A_2_B Ado receptors ameliorated colonic inflammation induced by DSS or 2,4,6-trinitrobenzene sulfonic acid (TNBS) (25). Moreover, also A_1_ Ado receptors may act proinflammatory by directly stimulating neutrophil adherence to endothelium and inducing chemotaxis towards inflammatory tissues (27). Thus, by showing that antagonists to A_2_B and A1 Ado receptors are able to suppress inflammation, one can conclude that Ado itself has proinflammatory functions, which may promote tumorigenesis at later stages of the disease, involving mechanisms as outlined before.

### 3.2 Effects of Ado on leukocytes in tumor and inflammation

#### 3.2.1 Macrophages

Macrophages are heterogenous myeloid cells originating from monocyte precursors in the blood that differentiate in the presence of cytokines and growth factors in the tissues they have infiltrated (28). Macrophages are present during chronic inflammation, during the development of malignant tumors and the progression of tumor growth (29). Classically activated M1 phenotype macrophages exhibit a pro-inflammatory phenotype and are present at sites of chronic inflammation during the early stages of cancer. The exact role of macrophages in early stages of cancer is controversial, since previous studies claimed that macrophages contribute to generating a milieu that promotes neoplasia by releasing copious amounts of mutagenic free radicals that promote cell transformation (30, 31). During the maintenance of inflammation, uncontrolled macrophage responses can become pathogenic and lead to disease progression and chronic inflammation (32). However, other studies added data supporting that M1 macrophages have inflammatory, but more predominantly, tumor-destructive phenotypes, as they eradicate only neo-transformed cells instead of normal cells (33–35). Moreover, they antagonize the tumor-promoting actions of suppressive cells (36).

By contrast, alternatively activated M2 macrophages display an anti-inflammatory phenotype, and comprise the main population when macrophages infiltrating established tumors. The term tumor-associated macrophages (TAMs) is frequently synonymously used. The polarization of TAMs, controlled by cancer cells, is not fixed to distinct M1 or M2 subpopulations, but a rather hybrid activation state of pro- and anti-inflammatory phenotype that can be found in developing cancers (28). Therefore, as cancer progresses, the malignant cells may hijack the polarization of macrophages which were initially recruited by an inflammatory response, by secreting M2-differentiating cytokines and chemokines, e.g. interleukin10 (IL10), CC chemokine ligand (CCL)2/3/4/5/7/8, CXC chemokine ligand (CXCL)12, vascular endothelial growth factor (VEGF), and Platelet-derived growth factor (PDGF). As a result, the M2-like macrophage population increases and appears to be the main population in later tumors (37).

In these processes, Ado has various inhibitory effects on macrophages, as it blocks their colony stimulating factor (M-CSF)-dependent proliferation (38), suppresses their phagocytic function (39), and dampens M1 macrophage activation mediated by A_2_A receptors (40). In addition, Ado promotes alternative-macrophage activation, as shown by the increased expression of several M2-macrophage markers, including arginase 1, tissue inhibitor of matrix metalloproteinase 1 and macrophage galactose-type C lectin 1. This is mainly mediated by the engagement of A_2_B Ado receptors and to a lesser extent by A_2_A receptors (41). Recent studies suggest a role of tumor-derived exosomes in promoting A_2_B Ado receptor-mediated polarization of macrophages toward an M2-like phenotype by carrying enzymatically active CD39/CD73 and Ado. The macrophages reprogrammed by tumor- derived exosomes secrete elevated concentration of pro-angiogenic factors (e.g. Angiopoietin-1, Endothelin-1, Platelet Factor 4 and Serpin E1) and subsequently stimulate growth of endothelial cells (42). Ado, generated by cervical cancer cells, stimulate the migration of myeloid cells to cancer tissues, in which they differentiate to CD39 and CD73-expressing M2-polarized macrophages. Thus, the M2-like macrophages contribute to raising extracellular concentrations of Ado and form a self-amplifying immunosuppressive mechanism (43). In the aggregate, these effects show synergistic actions of Ado and tumor derived factors, facilitating the conversion of proinflammatory M1 macrophages, which may initially be recruited by inflamed tissues, into tumor-permissive M2 subtypes (**Table 1**).

**Table 1:** Effects of adenosine on the function of different cells.

Cell Type	Adenosine-mediated effect on cells	Ref.
Macrophages	Dampens M1 proliferation and activity by A2A; Favours tumor-promoting M2 polarization	(40–42)
Dendritic cells	Inhibits DC maturation and activation; Induces expression of inhibitory molecule; Favours tolerogenic DC differentiation	(21, 44)
CD8^+^ T cells	Suppresses activation, proliferation and cytokine production; Upregulates co-inhibitory molecules	(45, 46)
CD4^+^ T cells	Suppresses cytokine production and expansion of Th1 and Th2	(47, 48)
Tregs	Promotes Tregs expansion, production of immuno-suppressive cytokines as well as expression of co-inhibitory receptors	(36, 49, 50)
Natural Killer cells	Hinders NK cell maturation, proliferation and cytotoxic function	(51)
Neutrophils	Suppresses adhesion, migration and effector functions	(52, 53)
B cells	Blocks BCR and TLR4 signalling and impairs the activation and survival of B cells	(54)
Fibroblasts	Promotes proliferation; Stimulates production of matrix proteins and collagen	(55–57)
Keratinocytes	Increases proliferation	(58)

#### 3.2.2 Dendritic cells

Dendritic cells (DCs) are myeloid cells that bridge innate immunity and adaptive immunity, by presenting antigen and activating T cells during infection and tumor pathogenesis (59). Thus, they are key players that are present in the tissues and lymphoid organs when the transitions from acute to chronic inflammation and finally to tumor generation takes place.

Ado, by engagement of A_2_B receptors (60), modifies DC maturation, as shown by reducing expression of MHC class II and CD86, as well as by reduction of tumor necrosis factor α (TNFα) and IL12 secretion, and by increased IL10 production (44, 61). Consequently, specific inhibition of A_2_B Ado receptors improves DC activation by increasing the production interferon γ (IFNγ) and the IFNγ-inducible chemokine CXCL10. It leads to enhanced recruitment of activated T cells that express CXCR3, the receptor for CXCL10, thereby reducing the growth of MB49 bladder- and 4T1 mammary carcinomas (62). A_2_A Ado receptors have direct suppressive effects on the function of tumor associated macrophages and DCs. This is facilitated by reducing IL-12 secretion and increasing IL-10 expression, leading to indirect suppression of T- and natural killer (NK) cells. In accordance with this, myeloid-specific deletion of A_2_A Ado receptors in mice led to enhanced effector function of DCs, T cells and NK cells, preventing them from developing primary and metastatic tumors (63).

Moreover, also A_2_B Ado receptors are active in DCs, as their engagement modifies the differentiation of DCs towards a phenotype lacking expression of the DC marker CD1a. Instead, these DCs display increased VEGF production and high levels of tolerogenic molecules, e.g. VEGF, IL-8, IL-6, IL-10, cyclooxygenase-2, TGFβ, and IDO (indoleamine 2,3-dioxygenase). These Ado-induced DCs possess impaired allostimulatory functions and support tumor vascularization, resulting in accelerated tumor growth in mice (44). This resembles the action(s) of Ado in the polarization of macrophages towards M2 phenotype, and since both macrophages and DCs are derived from monocytes, they may share similar intrinsic mechanisms, which are triggered by Ado receptors (**Table 1**).

#### 3.2.3 T cells

##### 3.2.3.1 CD8^+^ T cells

Antitumor CD8^+^ T cells express both A_2_A and A_2_B Ado receptors and exert anti-tumor effect mainly through the production of IFNγ. Several independent studies using Ado receptor gene-targeted mouse models or selective Ado receptor inhibitors (45, 64–67) have established that Ado, mediated by A_2_A and/or A_2_B Ado receptors, inhibits the anti-tumor activity of CD8^+^ T cells, supporting metastasis and neoangiogenesis in cancerous tissues. In detail, A_2_A Ado receptor signaling in CD8^+^ T cells dampens T cell receptor signaling by inhibiting activation of Notch1 (68). It suppresses effector functions of tumor infiltrating CD8^+^ T cells by increased protein kinase A (PKA) activation, leading to impairment of the mTORC1 (mammalian target of rapamycin complex 1) pathway (69). Thereby, Ado disrupts T cell activation, proliferation and cytokine production (70). A_2_A Ado receptor engagement also suppress T cell effector functions by upregulating the expression of immune-checkpoint molecules, including TIM3 (T cell immunoglobulin and mucin domain-containing protein 3) and PD-1 on CD8^+^ effector T cells (71). Of note, CD39^+^CD8^+^ T cells in chronic viral infections displayed high expression of PD1 and cytotoxic T-lymphocyte–associated antigen 4 (CTLA4) (72). Gene expression arrays as well as analysis of surface molecules revealed an exhausted phenotype of T cells. However, whether this impacts the function is less clear, but the strong correlation of CD39 expression with an exhausted phenotype of T cells observed in chronic inflammation corroborates our notion that chronic infection and tumor development may be bridged by Ado.

As for the regulation of Ado production, it is plausible that enhanced expression of CD39 and(or) of CD73 by T cells (as well as on tissue cells), contributes to generation of Ado in tissues of tumor and chronic infections, and thus the activation of A_2_A/A_2_B signaling through paracrine and/or autocrine mechanisms is responsible for inducing dysfunction in T cells. Despite the broad inhibitory effect of Ado on T cells, A_2_A signaling was also reported to protect T cells from activation-induced cell death (73) and to be important for the differentiation of T cells with memory phenotype (74, 75). These two effects may contribute to the transition from acute to chronic inflammation, because Ado may impede the termination of an acute inflammation by (i) preventing the activation-induced cell death of activated T cells, and (ii) by inducing enhanced differentiation of memory T cells. This may keep the inflammation ongoing as memory T cells are long lived and fast reactive as compared to naïve T cells.

##### 3.2.3.2 CD4^+^ T cells

Ado suppresses the effector functions of both, CD8^+^ and CD4^+^ T cells (76). Extensive studies using Ado receptor subtype-selective agonists and antagonists demonstrate that Ado attenuates inflammatory cytokine production in CD4^+^ T cells, primarily via the A_2_A receptor. In murine CD4^+^ T cells, TCR signaling increased the expression of A_2_A but not of A_2_B receptor mRNA. Accordingly, A_2_A receptor-selective agonists ATL146e and CGS21680 (CGS) exhibited a prominent inhibition of the release of IFN-γ (77) that is mediated by cAMP accumulation. Furthermore, Ado was found to substantially inhibit the production of IFN-γ and IL-2 in human melanoma-specific CD4^+^ T helper (Th) 1 cells, mediated via cAMP-activating PKA type I, as revealed by the application of CGS and the A_2_A Ado receptor-selective antagonist ZM241385 (47). *In vivo*, CGS administration reduced expansion of alloantigen specific Th1 cells, and the inhibition was abrogated by IL-2 therapy (78). Additionally, A_2_A Ado receptor mRNA expression in Th2 effector T cells increased following TCR stimulation. A_2_A Ado receptor stimulation suppressed the development of TCR-stimulated naïve T cells into Th2 cells, as indicated by decreased IL-4 secretion after CGS treatment in TCR-stimulated effector Th2 cells (48).

The effect of Ado on Th17 cells is controversial. Ado favors Th17 differentiation by acting via A_2_B receptors on DCs and stimulating production of IL-6 (79, 80).

In an autoimmune uveitis model, a nonselective Ado receptor agonist, applied shortly prior to onset of the disease inhibits the Th1 response and enhances the Th17 responses. In contrast, in an early stage of the already ongoing diseases injection of the same amount of Ado receptor agonist inhibits both Th1 and Th17 responses (81). Furthermore, A_2_A Ado receptor activation in naïve CD4^+^ T cells skews their differentiation away from Th1 effector cells toward the expansion of immune-suppressive regulatory T cells (Tregs), a subset of CD4^+^ T cells highly expressing CD25 and the forkhead transcription factor Foxp3, which play a vital role in immune suppression (36, 82).

Accumulation of Tregs in tumor microenvironment is frequent, as Tregs comprise the majority of tumor-infiltrating lymphocytes (TILs) at later stages of tumor progression in murine and human tumors (83, 84). Although Tregs possess different means for immune suppression, they are capable of synthesizing Ado by expressing high levels of the ectoenzymes CD39 and CD73, which provide a major source for Treg derived Ado. For example, Ado has been shown to be a major mediator of Treg-mediated immune suppression, which is critical for the downregulation of inflammatory reactions and for preventing immune reactions going overboard. That has been shown in models of inflammatory skin diseases, whereby Tregs devoid of Ado-producing CD73 are impaired in their immunosuppressive function (85). Consequently, tumor associated Tregs clearly promote tumor growth by Ado production. That has been established in several human tumors and in murine cancer models. (86–88). Of interest, Ado, via A_2_A Ado receptors, also feeds back on Tregs in a way as it promotes Treg cell expansion, the production of immunosuppressive cytokines (including TGFβ and IL10) and the expression of co-inhibitory receptors including PD-1, CTLA4 and Lymphocyte Activation Gene 3 (LAG3) (49, 50). Thus, Tregs and Ado may enter a self-sustaining cycle, starting in chronic inflammation and continuing during tumor growth (**Table 1**).

#### 3.2.4 Other leukocyte subpopulations

As Ado receptors are almost ubiquitously expressed by all types of immune cells, NK cells, neutrophilic granulocytes (neutrophils) as well as B cells are also susceptible to Ado. But their contribution to tumor development during inflammation is rather undefined and the role of Ado is quite often simply to suppress the immune function of these cells to help tumors grow. For the sake of completeness, however, the function of those different subtypes will be briefly described in the following.

Natural killer (NK), together with the effector CD8^+^ T cells are effector lymphocytes of the innate immune system and the adaptive immune system, respectively. NK cells form the first line of defense against various viral infections and tumors (89) and Ado plays a vital role in modulation of its effector function. Earlier studies found that adenosine inhibited NK cell function by interfering granule exocytosis (90) and by reducing the ability of NK cells to adhere to neoplastic cells (91). In particular, A_2_A Ado receptors are abundantly expressed by NK cells, and A_2_A receptor activation decreased NK cell maturation and cytotoxic functions *in vitro* (51), suppressed pro-inflammatory cytokines and inhibited granzyme B, perforin and FAS-Ligand mediated tumor cell lysis by NK cells (51, 92, 93).

In neutrophils, different Ado receptors serve various functions during inflammation. A_3_ Ado receptor signaling has been reported to be the key Ado receptor that facilitate neutrophil chemotaxis by controlling their trans-endothelial migration (57). In contrast, A_2_A Ado receptor activation was reported to suppress adhesion and migration of neutrophils, as well as their effector functions (52, 53). And finally, A_2_B receptors, which are also expressed by neutrophils, contribute to the maintenance of vascular integrity and attenuate neutrophil leakage into the inflamed tissue, as A_2_B Ado receptor knockout mice subjected to hypoxia exhibit increased tissue infiltration of neutrophils (94).

In B cells, Ado blocks the downstream NF-κB signaling of the B cell receptor and toll-like receptor 4 (TLR4) in an A_2_A-receptor/cAMP-dependent manner, thus impairing the activation and survival of these cells (54). Moreover, CD39^high^ B cells from human peripheral blood possess enzymatically active regulatory effects which vigorously produce Ado and mediate suppression of effector T cells by acting on A_2_A Ado receptors. Meanwhile, Ado generated by suppressive B cells activates the A_1_ and A_2_A Ado receptors on adjacent B cells, which generates an autocrine signaling, and in turn, enlarges the proliferation and functionality of these regulatory CD39^high^ B cells (95). However, although B cells are not noticed as major tumor infiltrating population, their capabilities to produce Ado and their presence during inflammatory reactions may add to an immunosuppressive and yet tumor permissive tissue environment (**Table 1**).

### 3.3 Effect of Adenosine on non-immune cells

In the further course of an inflammation, after the infection has been cleared, Ado has to support the re-establishment of tissue integrity and wound healing by promoting proliferation of tissue cells, such as fibroblasts and keratinocytes. To this effect it has been shown that agonists of the A_2_A and A_2_B Ado receptors stimulate production of matrix proteins in fibroblasts and affect differentiation into cells, which are critical for wound healing (55–57). This can even be therapeutically exploited, as topical application of an A_2_A Ado receptor agonist improves wound healing (96) and increases angiogenesis (97) by the production of VEGF (98) and the down-regulation of thrombospondin-1 (99), which acts as inhibitor of angiogenesis.

Ado has been shown to promote collagen production of fibroblasts, leading to scleroderma-like symptoms (57). According to mouse data, this is mediated by A_2_A Ado receptors, as A_2_A Ado receptor deficient fibroblasts failed to produce collagen in response to Ado. Scleroderma is considered as chronic inflammatory disease (100, 101) and in its course scleroderma patients have a higher risk for colorectal-, breast- and lung cancer (102, 103).

More evidence of an interconnection of Ado in chronic inflammation and tumor growth can be derived from a study in humans suffering from a genetic defect in the Ado inactivating enzyme Adenosine Deaminase (ADA). These patients have a higher chance of developing Dermatofibrosarcoma protuberans (DFSP), a rare malignant skin tumor (104).

In addition to fibroblasts, also keratinocytes can react to stimulation by Ado with proliferation. Evidence is provided by investigations showing that keratinocytes undergo increased proliferation after engagement of A_2_A Ado receptors and an altered expression pattern of A_2_A Ado receptors is thought to play a role in the development of psoriasis (58). Psoriasis is a sever chronic inflammation of the skin, which is furthermore connected to an increased occurrence of keratinocyte cancer (105).

In mice, direct tumorigenic actions of Ado can be investigated much more precisely, as mouse lines with genetic defects tailored to ablate molecules involved in Ado-mediated signaling, can be produced. As for mesenchymal, i.e. fibroblast-derived, tumors it has been shown that the general carcinogenesis is impaired in mice lacking the major Ado producing ectoenzyme CD73 (106) and that ablation of the A_2_A Ado receptor or injection of its antagonist caffeine, suppressed the carcinogen-induced tumorigenesis (107). In mice there are many more studies on how Ado and respective antagonists can prevent tumor growth, but this is beyond the scope of this review and details can be found in our previous review (108).

## 4 Hypoxia as a common denominator between Adenosine, inflammation and tumor growth

**Figure 3:**
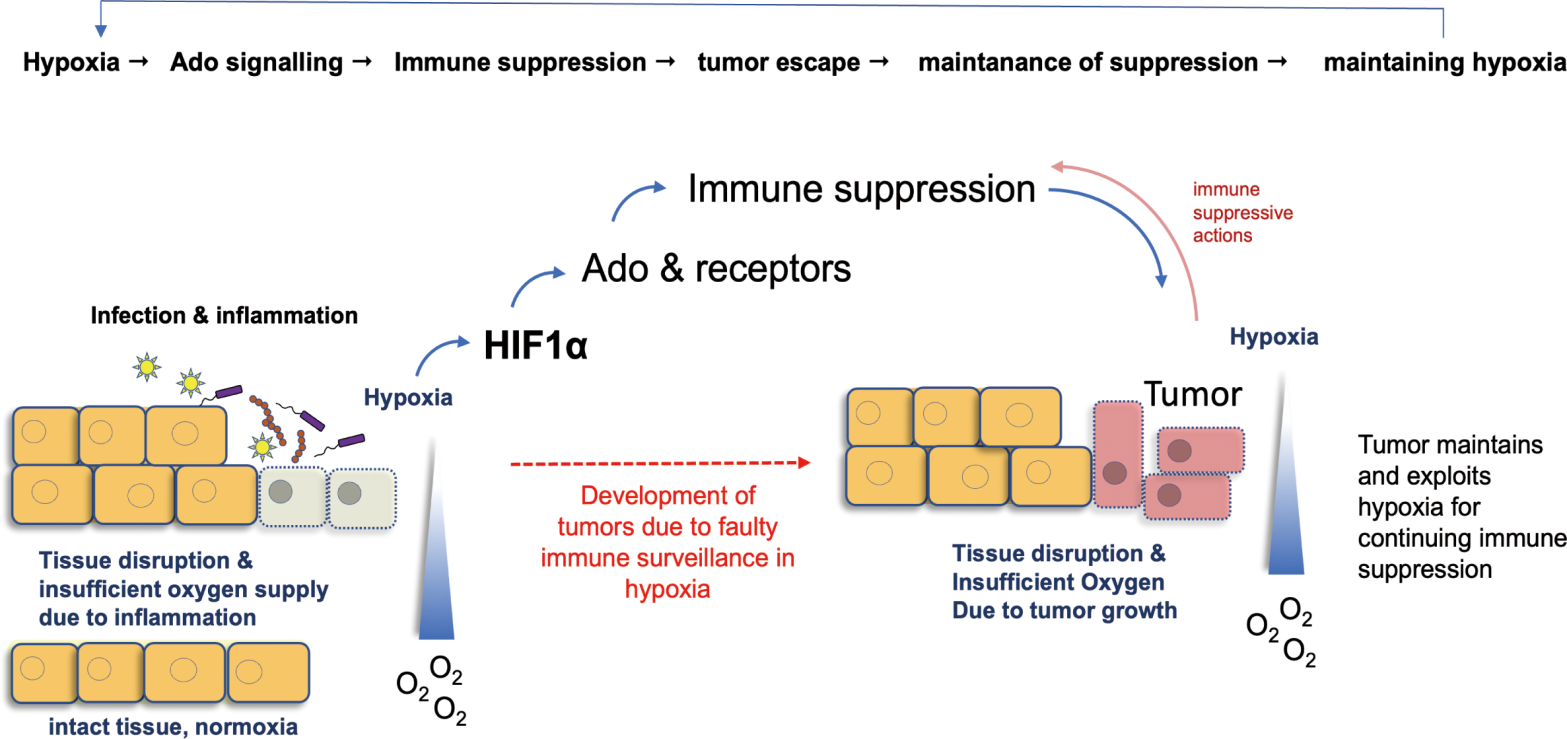
Hypoxia as a common denominator in Ado-induced mechanisms of tumor growth. Hypoxia, i.e. a reduced availability of oxygen, is a key event in inflammation. Mainly via HIF1α, it stimulates Ado production and differentiation of M2 macrophages. These events exert immune suppressive actions and hamper immunity of the body. Consequently, development of tumors from infected tissues can escape immune surveillance and growing cancers maintain hypoxia, which in turn stabilizes the immune suppressive actions of Ado. Ado: adenosine; HIF1α: hypoxia-inducible factor 1 α.

Findings have shown that the extracellular concentration of Ado in extracellular fluids of solid carcinomas may reach to 10^-4^ M (10 to 20-fold higher than normal concentration) (109). The accumulation of Ado in tumor microenvironments is probably due to a reduction in oxygen levels (hypoxia), which is common in cancer. It results from the fast growth of an expanding carcinoma outcompeting the development of a supportive vascular bed (110). For example, the hypoxic fraction in squamous cell carcinomas of the cervix and head and neck can be as high as 20-32% (111) and a connection to Ado can be delineated by results obtained with hypoxic cultures of 3LL Lewis lung carcinoma cells that have been shown to generate elevated levels of extracellular Ado (112). Notably, the extracellular Ado levels in tumors can be supplemented by the ectoenzymes CD39 and CD73 that additionally mediate production of Ado. The respective genes are induced by hypoxic situations (113, 114) and in the pathways the hypoxia-inducible factor1 alpha (HIF1α) is involved. It upregulates CD73 activity and subsequently increases synthesis of Ado (115, 116). Vice versa, blockade of CD73 or respective Ado receptors is able to promote normoxia in some cancer models (117, 118), suggesting a feedback mechanism that further strengthens a proposed Ado-hypoxia interconnection (**Figure 3**).

In terms of hypoxia, the tumor microenvironment can be considered as a chronic low-grade inflammation. These hypoxic tissue conditions are common in inflammation as well as during tumor growth, and a relation to “adenosinerg” signaling became evident early on, as conditions of low oxygen or inflammation favor the release of extracellular ATP/ADP (119, 120). This assumption has now been broadened by ample evidence showing that Ado metabolism and gene expression are tightly linked with oxygen signaling (121–125).

On a molecular level the relation between oxygen shortness and Ado became clear, after studies of Synnestvedt et al. (126) identified a binding site for HIF1α, the major signaling molecules in hypoxia, in the hypoxia response element promoter of the CD73 gene. In support of this, it was shown that CD73-deficient mice, i.e. mice impaired in producing extracellular Ado, suffer substantial vascular leakage and increased accumulation of lymphocytes when exposed to low oxygen (127). CD39, another surface molecule involved in Ado production, is induced in hypoxia by the transcription factor specificity protein 1 (Sp1) (123), which belongs to a hypoxia-induced gen set and has been shown to play a protective role in regulation of CD39 during cardiac and hepatic ischemia (128, 129). And finally, yet another enzyme involved in Ado turnover is affected by HIFs: the adenosine kinase. This enzyme converts Ado to Adenosine-monophosphate and is blocked by HIFs, which leads to a shift towards more Ado (as compared to Adenosine-monophosphate) in cells (130).

In addition to the production of Ado by enzymes such as ectonucleotidases, Ado concentrations are also directly influenced by HIFs, as HIF affects the transport of Ado by equilibrative nucleoside transporters (ENTs) and its G-protein-coupled receptors. For example, ENT1 and ENT2 (131, 132), two transporters that mediate uptake of Ado into cells, are downregulated by HIFs and therefore extracellular Ado will be increased. Finally, HIFs also affect the receptors for Ado, as for the A_2_A Ado receptor, it has been shown to be a target gene of HIF2α in human lung endothelial cells (133), while the A_2_B Ado receptor has been identified as a target gene of HIF1α (134, 135).

More experimental evidence supports the hypothesis that Ado promotes angiogenesis by stimulating VEGF production through engagement of A_2_A receptors (29). Synergistic up-regulation of VEGF expression is induced by Ado via A_2_A Ado receptors, together with endotoxin (98, 136) and(or) other toll-like receptors agonists (137, 138). As VEGF is a target of HIF1, several studies support that A_2_A Ado receptor activation stimulates VEGF production by inducing massive HIF1 expression in macrophages (98, 139), both of which are main events in response to hypoxia.

Hypoxia also appears to be a key driver in recruiting and modifying macrophages in tumor tissues. Hypoxia attracts macrophages by chemokines, HIF1/2 and endothelin-2 (140), and increases their angiogenic activity (141) by inducing high levels of pro-angiogenic factors such as VEGF and TNFα (142). The transition of M1 to M2 phenotype, is an effective method to permit the resolution of inflammation. However, M2 macrophages have a tumor permissive phenotype by contributing to various aspects of metastasis (as outlined in the previous chapter). They promote angiogenesis and cell proliferation, induce the local suppression of lymphocyte-mediated anti-tumor immunity and facilitate matrix deposition and remodeling (143).

In a nutshell, one can envision interconnected feedback loops of inflammation, Ado, hypoxia and tumor development. The primary role of Ado during inflammation is to harness over boarding immune activation and cells may sense an inflammatory environment by hypoxic conditions. In this feedback loop hypoxia leads to enhanced production of Ado that typically ameliorates inflammation. As a consequence, normoxic conditions will be reestablished and in the following normoxic conditions will lead to downregulation of Ado production.

However, production of Ado and Ado-mediated immune regulation takes time and/or may be not very effective as leukocytes and tissue cells differentially express Ado receptors. Therefore, inflammation may not be fully terminated by Ado and a lingering (i.e. chronic) inflammation maintains a hypoxic environment, keeping Ado concentrations elevated. Now, a self-sustaining loop is keeping two immunosuppressive mechanisms (i.e. Ado and Hypoxia) active and neoplasm-inducing conditions will arise.

Once a tumor grows, hypoxia is maintained by the tumor itself, independent from the inflammation. This may further stimulate Ado production, but as the tumor causes hypoxia and not the infiltrating leukocytes, the regulatory feedback loop between Hypoxia, Ado and inflammation is disrupted. The tumor can now profit from the suppressive tissue environment and escape immune surveillance.

## 5 Conclusion

In the course of an inflammation the potent immune suppressor Ado is produced by cells to prevent overshooting inflammation and to induce healing of the tissue. At the same time inflammation causes massive mutations and stimulates extensive cell proliferation that requires active immune surveillance to prevent induction of tumors. If this commonly accepted and fine-tuned immunosuppression by Ado is out of balance, for example by chronic and prolonged inflammation, immune suppressive actions of Ado may outcompete the beneficial “healing” and tissue remodeling capacities of Ado, and inflammation-driven mutations may easily lead to tumors that can escape the immune surveillance, which is suppressed by Ado.

## Author contributions

LC: Writing – original draft, Writing – review & editing. MA: Writing – review & editing. KM: Writing – review & editing.

## Glossary

**Table:**

ADA	adenosine deaminase
Ado	adenosine
ADP	adenosien diphosphate
AID	activation-induced cytidine deaminase
AMP	adenosine monophosphate
ARs	adenosine receptors
ATP	adenosine triphosphate
Bax	Bcl-2 associated X protein
Bcl-6	B-cell lymphoma 6
CCL	CC chemokine ligand
CGS	CGS21680
CTLA-4	cytotoxic T-lymphocyte-associated antigen 4
CXCL	CXC chemokine ligand
CXCR	CXC chemokine receptor
DC	dendritic cell
DFSP	aermatofibrosarcoma protuberans
DSS	dextran sodium sulfate
ENTs	nucleoside transporters
HIF	hypoxia-inducible factor
IDO	indoleamine 2,3-dioxygenase
IFNγ	interferon γ
IL	interleukin
Jmjd3	Jumonji domain-containing protein D3
LAG3	Lymphocyte Activation Gene 3
mTORC1	mammalian target of rapamycin complex 1
NK	natural killer
PD-1	programmed death-1
PD-L2	programmed death-ligand 2
PDGF	Platelet-derived growth factor
PKA	protein kinase A
ROS	reactive oxygen species
Sp1	specificity protein 1
TAMs	tumor-associated macrophages
Tgfbr2	TGF-β receptor type 2
TGFβ	Transforming growth factor β
TIM3	T cell immunoglobulin and mucin domain-containing protein 3
TNBS	2,4,6-trinitrobenzene sulfonic acid
TNF	tumor necrosis factor
Tregs	regulatory T cells
VEGF	vascular endothelial growth factor
